# Metabolic Aspects of Lentil–*Fusarium* Interactions

**DOI:** 10.3390/plants13142005

**Published:** 2024-07-22

**Authors:** Chrysanthi Foti, Antonios Zambounis, Evmorfia P. Bataka, Chrysanthi Kalloniati, Evangelia Panagiotaki, Christos T. Nakas, Emmanouil Flemetakis, Ourania I. Pavli

**Affiliations:** 1 Laboratory of Plant Breeding, Department of Agriculture, Crop Production and Rural Environment, University of Thessaly, Fytokou St., 384 46 Volos, Greece; chrysafoti@yahoo.gr (C.F.); evpanag@uth.gr (E.P.); 2 Hellenic Agricultural Organization-DIMITRA (ELGO-DIMITRA), Institute of Plant Breeding and Genetic Resources, 570 01 Thessaloniki, Greece; azampounis@elgo.gr; 3 Laboratory of Biometry, Department of Agriculture, Crop Production and Rural Environment, University of Thessaly, Fytokou St., 384 46 Volos, Greece; bataka@uth.gr (E.P.B.); cnakas@uth.gr (C.T.N.); 4 Laboratory of Molecular Biology, Department of Biotechnology, School of Applied Biology and Biotechnology, Agricultural University of Athens, Iera Odos 75, 11855 Athens, Greece; xkalloni@gmail.com (C.K.); mflem@aua.gr (E.F.); 5 Department of Clinical Chemistry, Inselspital, Bern University Hospital, University of Bern, 3010 Bern, Switzerland

**Keywords:** lentil, *Fusarium oxysporum* f. sp. *lentis*, *Fol*-inoculation, metabolic response, GC-MS, metabolic biomarkers, *Fol*-resistance, early selection, resistance breeding

## Abstract

*Fusarium oxysporum* f. sp. *lentis* (*Fol*) is considered the most destructive disease for lentil (*Lens culinaris* Medik.) worldwide. Despite the extensive studies elucidating plants’ metabolic response to fungal agents, there is a knowledge gap in the biochemical mechanisms governing *Fol*-resistance in lentil. Τhis study aimed at comparatively evaluating the metabolic response of two lentil genotypes, with contrasting phenotypes for *Fol*-resistance, to *Fol*-inoculation. Apart from gaining insights into the metabolic reprogramming in response to *Fol*-inoculation, the study focused on discovering novel biomarkers to improve early selection for *Fol*-resistance. GC-MS-mediated metabolic profiling of leaves and roots was employed to monitor changes across genotypes and treatments as well as their interaction. In total, the analysis yielded 178 quantifiable compounds, of which the vast majority belonged to the groups of carbohydrates, amino acids, polyols and organic acids. Despite the magnitude of metabolic fluctuations in response to *Fol*-inoculation in both genotypes under study, significant alterations were noted in the content of 18 compounds, of which 10 and 8 compounds referred to roots and shoots, respectively. Overall data underline the crucial contribution of palatinitol and L-proline in the metabolic response of roots and shoots, respectively, thus offering possibilities for their exploitation as metabolic biomarkers for *Fol*-resistance in lentil. To the best of our knowledge, this is the first metabolomics-based approach to unraveling the effects of *Fol*-inoculation on lentil’s metabolome, thus providing crucial information related to key aspects of lentil–*Fol* interaction. Future investigations in metabolic aspects of lentil–*Fol* interactions will undoubtedly revolutionize the search for metabolites underlying *Fol*-resistance, thus paving the way towards upgrading breeding efforts to combat fusarium wilt in lentil.

## 1. Introduction

Lentil is an ancient legume, domesticated about 10,000 years ago [1], which is currently cultivated worldwide and is recognized as one of the most nutritious pulse crops. Lentil seeds supply protein, fiber and essential micronutrients to human and animal nutrition, while the crop’s inclusion into rotation systems with cereals offers possibilities of enriching the soil via nitrogen fixation and carbon sequestration [2]. Global lentil production is severely challenged by a series of fungal diseases [3], of which Fusarium wilt, caused by *Fusarium oxysporum* f. sp. *lentis* (*Fol*) [4,5,6,7,8,9], is considered the most destructive. In lentil, wilt disease causes yield losses up to 50% or even complete crop loss, while the disease epidemics strongly depend on genotype, crop stage—early wilt or late wilt—and agroclimatic conditions [10]. *Fol*-infection is mediated by root entrance and growth in the plant xylem, affecting the vascular system and preventing water and nutrient supply, ultimately leading to severe wilting, discoloration and plant death [9]. The pathogen produces (i) microconidia, which are ovoid- or kidney-shaped, hyaline and often single-celled, (ii) two- to seven-celled, long macroconidia and (iii) single-celled chlamydospores, which are oval- or spherical-shaped and formed singly in macroconidia or apical or intercalary in the hyphae [7,9]. The pathogen overwinters for several years in the soil and on crop residues of infected plants as chlamydospores or mycelium, while primary infection is mediated either by soil-borne inoculum, in the form of chlamydospores and/or dormant mycelium, or infected straws and seeds [7].

Taking into account that disease management is particularly difficult, owing to the long-term persistence of chlamydospores in the soil [5] as well as the lack of efficient agronomic and chemical practices, the most cost-effective and sustainable means of minimizing crop losses is through the use of varieties enriched with *Fol*-resistance [7,11,12,13,14]. Towards this direction, several studies revealed the existence of significant genetic variation for *Fol*-resistance [11,15]. In relevant breeding programs, selection for *Fol*-resistance is most commonly practiced under field conditions with naturally occurring levels of inoculum [16,17]. The identification of resistance sources is routinely employed via wilt sick plot (WSP) evaluation as it allows for simultaneous screening of a large number of germplasm materials [5,9,11,18,19,20]. Although offering comparative advantages over other laborious and time-consuming screening approaches, the effectiveness of WSP is compromised by the polygenic nature of resistance and the fact that under field conditions plants are simultaneously challenged by a wide spectrum of coexisting stress stimuli. An alternative approach to overcoming such limitations involved *Fol*-inoculation and genotype evaluation under controlled conditions [4,10,16,17,21]. Despite the knowledge so far acquired regarding the molecular responses to fusarium wilt disease in a plethora of plant species, there is a knowledge gap in the molecular aspects of *Fol*–lentil interactions as well as the mechanisms governing *Fol*-resistance [7,14,22,23,24,25]. 

Aiming at upgrading the efficacy of selection procedures for *Fol*-resistance, the emphasis will be undoubtedly placed on applying the available advanced genomic tools, including high-throughput omics technologies, in crop tolerance improvement. Metabolomics offer possibilities of gaining in-depth knowledge related to the global regulatory networks controlling stress resistance as well as for pinpointing specific metabolic compounds which may serve as biomarkers for indirect screening for resistance, ideally at early growth stages. Given that metabolomics enable simultaneous quantification of a large pool of metabolites that fluctuate in response to various stimuli, investigating the host–microbe interactions through changes at the metabolome provides possibilities of advancing knowledge on both pathogenicity and plant metabolic responses to the disease. In this context, metabolomics research of plant–fungal interactions is progressively gaining ground as a systems biology tool to develop novel control strategies for fungal diseases [26]. In legumes, the research conducted has placed emphasis on a wide range of biological systems, including *Colletotrichum*–lupine [27,28], *Sclerotinia*–bean [29], *Rhizoctonia*–soybean [30] and *Sclerotinia*–soybean [31]. Relevant studies for species of Fusarium refer to the biological systems of *F. graminearum*–cereals [32,33,34] and *F. tucumaniae*–soybean [35], while the reports regarding *F. oxysporum* are limited to chickpea [23,36].

Given the widespread occurrence of *Fol* and the severe yield and economic penalties on lentil crop as well as the lack of molecular data related to the *Fol*–lentil pathosystem, this study aimed at comparatively assessing the metabolic response of two lentil genotypes, showing contrasting resistance phenotypes, to *Fol*-inoculation. Overall findings are anticipated to provide insights into the molecular underpinnings of *Fol*-resistance and contribute towards pinpointing the pool of key metabolites that may serve as biomarkers for selection procedures.

## 2. Results

### 2.1. Comparative Metabolic Response of Contrasting Genotypes to Fol-Inoculation

The leaf and root metabolic profiles of two lentil genotypes, with contrasting phenotypes for *Fol*-resistance, were comparatively assessed following *Fol*-inoculation. Changes in metabolite accumulation in both shoots and roots were expressed as a relative response ratio of *Fol*-inoculated seedlings to controls (*Fol*/C). 

**Figure 1:**
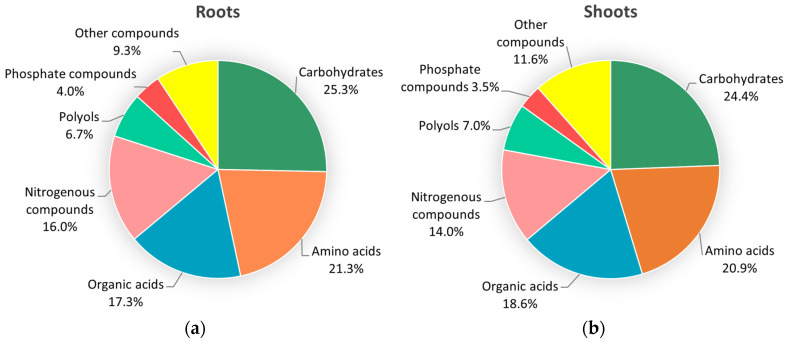
Classification of identified metabolites, applying GC-MS metabolomics analysis, in roots (**a**) and shoots (**b**) of lentil seedlings following *Fol*-inoculation.

In total, the analysis yielded 178 quantifiable compounds, of which 75 and 86 metabolites for roots and shoots, respectively, showed highly reproducible patterns among biological replications (*n* = 4) per genotype–treatment combination, and, therefore, these compounds were included in the analysis (Tables S1 and S2). In roots, the identified metabolites were distributed to the chemical groups of carbohydrates (25.3%), amino acids (21.3%), organic acids (17.3%), nitrogenous compounds (16%), polyols (6.7%) and phosphate compounds (4%), and a portion of them belonged to other chemical groups (9.3%) (Figure 1a). In shoots, the vast majority of the identified metabolites belonged to the group of carbohydrates (24.4%), amino acids (20.9%), organic acids (18.6%), nitrogenous compounds (14%), polyols (7%) and phosphate compounds (3.5%), while a small portion of them was classified into other groups (11.6%) (Figure 1b).

**Figure 2:**
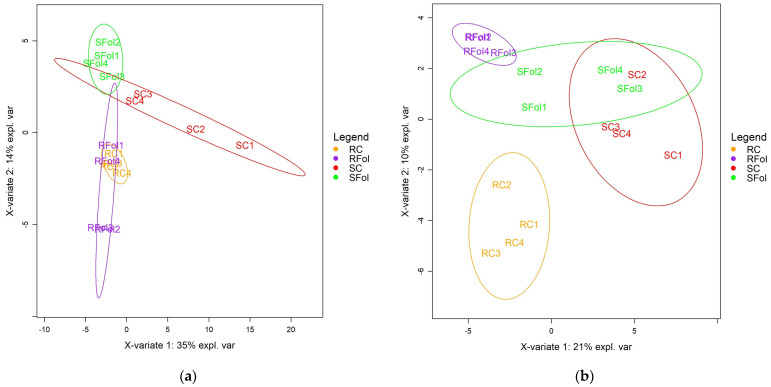
Partial least squares–discriminant analysis (PLS-DA). PC1/PC2 score plots for the GC-MS metabolic profiles of lentil roots (**a**) and shoots (**b**). R: resistant genotype (ILL-590), S: susceptible genotype (ILL-6031), C: control seedlings, Fol: *Fol*-inoculated seedlings. Four pooled samples were used for each genotype–treatment combination (*n* = 4).

Partial least squares–discriminant analysis (PLS-DA) was applied to discover relationships between the metabolic profiles of control and *Fol*-inoculated seedlings as well as between ILL-590 and ILL-6031 genotypes. The analysis revealed a differential response of contrasting genotypes but also a discrimination between control and *Fol*-inoculated seedlings. The PLS-DA score plots for roots revealed a satisfactory separation between ILL-590 and ILL-6031, while a partial overlap was noted in the metabolic response of control and *Fol*-inoculated seedlings. According to PC1 (35%), roots displayed a similar metabolic response and no clear distinction into separate groups was obtained. PC2 (14%) provided a moderate separation between contrasting genotypes, yet a metabolic response overlap between control and *Fol*-inoculated seedlings was noted for both genotypes (Figure 2a). For shoots, the PLS-DA score plots discriminated the metabolic profiles of contrasting genotypes as well as those of control and *Fol*-inoculated seedlings, yet the most profound differences referred to control and *Fol*-inoculated seedlings of ILL-590. PC1 (21%) did not show a clear separation into distinct groups, with the exception of the control seedlings of ILL-6031 which were clearly distinguished from control and *Fol*-inoculated seedlings of ILL-590. However, PC2 (10%) revealed a discrimination between contrasting genotypes and further clearly distinguished control and *Fol*-inoculated seedlings of ILL-590. In contrast, shoots of ILL-6031 displayed a metabolic response overlap at control and *Fol*-inoculated seedlings and no clear distinction into separate groups was obtained (Figure 2b).

### 2.2. Global Fluctuations in the Metabolome of Contrasting Genotypes in Response to Fol-Inoculation

In response to *Fol*-inoculation, lentil’s root and shoot metabolic profiles were significantly affected in both ILL-590 and ILL-6031. Despite the magnitude of the observed metabolic fluctuations, the analysis revealed that the content of 10 and 8 compounds was significantly altered in roots and shoots, respectively. The significance of changes was determined on the basis of the *p*-value criterion (*p* < 0.05) with reference at the level of genotype (G) and/or treatment (T) and/or G × T interaction. The largest pool of compounds with altered metabolic content belongs to the chemical groups of carbohydrates, amino acids, polyols, organic acids, nitrogenous compounds, carboxylic acids and phosphate compounds. Such findings underline that lentil’s response to *Fol*-inoculation involves metabolic adjustments in primary and secondary metabolism. The most substantial changes in the metabolic content of roots and shoots of control and *Fol*-inoculated seedlings of ILL-590 and ILL-6031 are listed in Table 1.

Below, lentil’s metabolic response to *Fol*-inoculation is described with reference to the chemical groups of metabolic compounds with mostly altered content in roots and shoots of control and *Fol*-inoculated seedlings of ILL-590 and ILL-6031.

#### 2.2.1. Carbohydrates

In response to *Fol*-inoculation, the metabolic content of carbohydrates was significantly affected in the roots, while in the shoots no substantial changes were noted. For roots, in total 19 metabolites were analyzed, of which four metabolites, namely, cellobiose, leucrose, D-trehalose and talose, presented significantly altered content between genotypes and/or treatments and/or their interaction. Addressing differences between genotypes, cellobiose presented an opposite accumulation pattern in contrasting genotypes, with ILL-590 showing an increase and ILL-6031 a decrease in *Fol*-inoculated seedlings. Further, leucrose and talose were exclusively detected in controls and were depleted in *Fol*-inoculated seedlings of both genotypes, the latter also presenting a significant difference at the level of treatment and G × T interaction. Finally, D-trehalose was increased in *Fol*-inoculated seedlings of both genotypes, with the increase being significant only in ILL-590 (~3 fold).

#### 2.2.2. Amino Acids

In the chemical group of amino acids, 16 and 18 metabolites were analyzed in roots and shoots, while substantial changes were noted in the accumulation of one and two compounds for roots and shoots, respectively. As far as roots are concerned, only L-ornithine differed significantly between treatments, presenting an exclusive accumulation in *Fol*-inoculated seedlings of both genotypes. Despite the absence of statistically significant fluctuations, the metabolic profile of roots was further marked by a profound increase in the content of L-asparagine in *Fol*-inoculated seedlings of both genotypes. In particular, the response to *Fol*-inoculation involved an increase in the content of L-asparagine, ranging from ~11 up to ~34 fold in ILL-590 and ILL-6031, respectively.

In relation to the shoot metabolic response to *Fol*-inoculation, the analysis pointed to the significance of changes for the accumulation patterns of L-proline and L-asparagine. More importantly, L-proline showed an opposite accumulation pattern in contrasting genotypes, which involved an increased and decreased content in the *Fol*-inoculated seedlings of ILL-590 and ILL-6031, respectively. In contrast, L-asparagine showed a cumulative metabolic response to *Fol*-inoculation in both genotypes, which was marked by a profound increase in ILL-590 (up to ~23 fold). 

#### 2.2.3. Polyols

The metabolic content of polyols was affected in both roots and shoots, exhibiting accumulation patterns which differed significantly either between genotypes or between treatments. In roots, five metabolites were included in the analysis, of which palatinitol and arabitol were significantly altered. The content of palatinitol was increased in *Fol*-inoculated seedlings of both genotypes, yet it differed significantly both at the genotype and the treatment level. In contrast, the content of arabitol differed between contrasting genotypes, showing an increase and decrease in *Fol*-inoculated seedlings of ILL-590 and ILL-6031, respectively.

In shoots, six metabolites were analyzed, while substantial changes were noted in the accumulation of acetol and 2-butyne-1,4-diol. Both compounds showed a differential response between genotypes, with ILL-590 showing zero accumulation in both *Fol*-inoculated and control seedlings and ILL-6031 presenting a decreased accumulation in *Fol*-inoculated seedlings as compared to the respective controls. Further, the metabolic response to *Fol* involved fluctuations in arabitol content, which were mainly marked by its increase in shoots of ILL-590 (~4 fold).

#### 2.2.4. Organic Acids

In the group of organic acids, despite the observed variation referring to a large pool of metabolites in both roots and shoots, significant alteration was noted to the content of a small portion of them. In roots, 13 metabolic compounds were analyzed, while substantial fluctuations were noted in the accumulation of meleamic acid. Although presenting a decreasing trend in response to *Fol*-inoculation in both genotypes, the content of meleamic acid differed significantly both between genotypes and treatments. 

In shoots, in total, 15 metabolites were included in the analysis, yet significant changes were recorded in the content of fumaric acid and iminodiaseic acid. As such, fumaric acid differed significantly at the level of genotype, treatment and G × T interaction, showing zero accumulation in both *Fol*-inoculated and control seedlings of ILL-590 and a substantial decrease in *Fol*-inoculated seedlings of ILL-6031. In relation to iminodiacetic acid, although showing a decrease in *Fol*-inoculated seedlings of both genotypes, its content differed significantly between ILL-590 and ILL-6031.

#### 2.2.5. Carboxylic Acids

In the group of carboxylic acids, substantial changes were only recorded in the shoot content of 3-hydroxypropanoic acid which differed significantly between genotypes, treatments and their interaction. Specifically, ILL-590 showed zero accumulation in both *Fol*-inoculated and control seedlings, while in ILL-6031 3-hydroxypropanoic acid was exclusively detected in control seedlings. 

#### 2.2.6. Nitrogenous Compounds

As far as nitrogenous compounds are concerned, significant differences were only recorded in the roots. Specifically, 12 metabolic compounds were analyzed, of which 2-amino-1-phenylethhanol and putrescine were significantly altered. Both amines differed between *Fol*-inoculated and control seedlings, while putrescine also differed between genotypes and G × T interaction. In particular, 2-amino-1-phenylethanol was significantly increased in *Fol*-inoculated seedlings of both genotypes, yet it showed a marked increase in ILL-590 (~12 fold). In contrast to the abovementioned cumulative pattern, putrescine was significantly reduced in *Fol*-inoculated seedlings of both genotypes while presenting zero accumulation in *Fol*-infected roots of ILL-6031.

Interestingly, although not presenting statistically significant fluctuations, the shoot metabolic response to *Fol* involved an increased content of 2-amino-1-phenylethanol in both genotypes, which was marked by its drastic increase in ILL-590 (~20 fold). 

#### 2.2.7. Phosphate Compounds

In relation to phosphate compounds, a significant alteration was only recorded in the shoot metabolic content of glycerol 1-phosphate. Specifically, glycerol 1-phosphate differed significantly between treatments, thus suggesting its contribution to the *Fol*-attributed metabolic responses. Although showing a cumulative pattern in shoots of both genotypes, glycerol 1-phosphate was depleted in controls and was exclusively detected in *Fol*-inoculated seedlings of ILL-590, while it was slightly increased in *Fol*-inoculated seedlings of ILL-6031.

**Figure 3:**
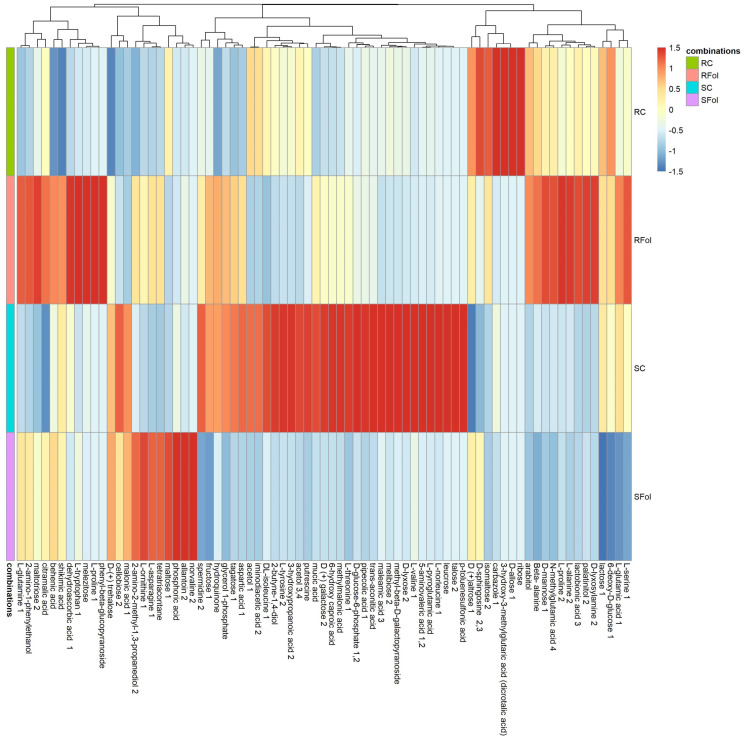
A heat map with customized metabolomic data visualization for compounds detected in lentil roots. Each column represents a metabolite feature, and each row represents the median of 4 replicates for each genotype–treatment combination (*n* = 4). Metabolite features were projected on the heat map and used for sample clustering. The scaled expression value of each feature is plotted in red–blue color scale. The red and blue colors indicate accumulated and depleted content, respectively. R: resistant genotype (ILL-590), S: susceptible genotype (ILL-6031), C: control seedlings, Fol: *Fol*-inoculated seedlings.

In order to visualize the effects of *Fol*-inoculation in the metabolome of ILL-590 and ILL-6031, heat maps were constructed for both root and shoot metabolic profiles, using a median of 4 replicates (*n* = 4) for each genotype–treatment combination. For roots, hierarchical clustering analysis confirmed three distinct major clusters defined by the diversity of metabolic patterns across genotypes and treatments, while also depicting differences in the metabolic content of certain compounds. The majority of compounds with major contribution to metabolic divergence were classified in the groups of carbohydrates, polyols and amino acids (Figure 3).

**Figure 4:**
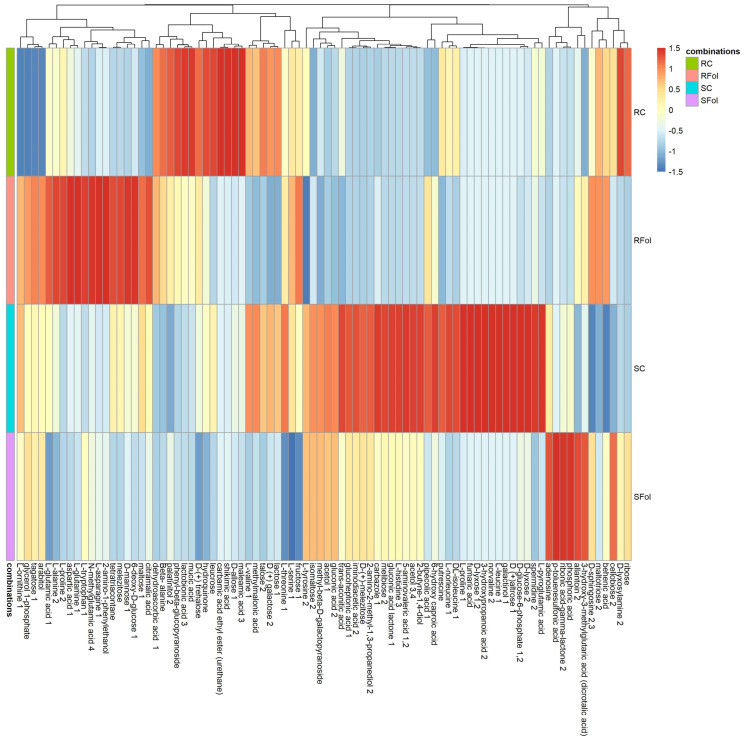
A heat map with customized metabolomic data visualization for compounds detected in lentil shoots. Each column represents a metabolite feature, and each row represents the median of 4 replicates for each genotype–treatment combination (*n* = 4). Metabolite features were projected on the heat map and used for sample clustering. The scaled expression value of each feature is plotted in red–blue color scale. The red and blue colors indicate accumulated and depleted content, respectively. R: resistant genotype (ILL-590), S: susceptible genotype (ILL-6031), C: control seedlings, Fol: *Fol*-inoculated seedlings.

For shoots, HCA identified four distinct clusters with similar metabolic patterns as well as groups of discriminating metabolites that contribute to clustering. The observed clusters highlight the differential metabolic response of ILL-590 and ILL-6031 to *Fol*-inoculation, thus providing opportunities for pinpointing distinctive metabolic patterns associated with either *Fol*-resistance or *Fol*-susceptibility. Addressing the differential response of ILL-590 and ILL-6031 to *Fol*-inoculation, the vast majority of discriminating metabolites belong to the class of carbohydrates, organic acids, amino acids and polyols (Figure 4).

### 2.3. Exploring the Metabolic Response to Fol-Inoculation Based on an XGBoost Prediction Model

**Figure 5:**
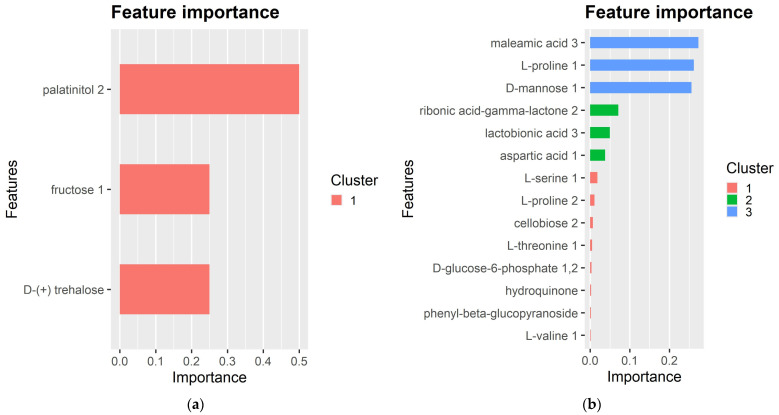
Feature importance bar plot. Importance ranking of metabolic compounds that are major contributors to lentil’s response to *Fol*-inoculation in roots (**a**) and shoots (**b**), according to extreme gradient boosting modeling.

Aiming at pinpointing compounds that are major contributors to lentil’s response to *Fol*-inoculation, metabolomics data were combined with a machine learning model (XGBoost). In this context, the gradient boosting methodology was applied to create a prediction model consisting of the variables that contribute strongly to prediction. The top-ranked predictors are presented in Figure 5 (Table S3). For roots, the XGBoost model revealed that palatinitol, D-trehalose and fructose are the most important contributors to lentil’s response to *Fol*-inoculation. The analysis of shoot metabolic profiles provides evidence related to the significant contribution of maleamic acid, L-proline, D-mannose, ribonic acid-g-lactone, lactobionic acid, aspartic acid, L-serine, L-proline, cellobiose, D-glucose-6-phosphate, hydroquinone, phenyl-b-glycopyranoside and L-valine, the former three compounds providing higher relative contributions as evidenced by the importance metrics (Table S3). 

## 3. Discussion

Metabolomics analysis has undoubtedly become an essential analytical tool that yields novel insights into the complex networks regulating plants’ metabolic response to various stimuli. Addressing the elucidation of biochemical mechanisms and cellular metabolic processes involved in defense responses and the comparative analysis of the metabolic profiles of genotypes with contrasting-resistance phenotypes provides powerful knowledge, while also offering opportunities for the discovery of new biomarkers [37,38]. During host–pathogen interactions, plants mount a series of multi-layered responses, often involving a plethora of compounds, whose identification is a key step for the selection and/or development of resistant cultivars [31]. 

In legumes, numerous studies have been carried out to better understand how plants metabolically respond to various pathogens, the majority of them being focused on model plants [23]. Specifically in lentil, metabolomics has been employed to gain insights into the metabolic responses to drought and salinity stresses [39,40,41], yet very few studies investigate its interaction with devastating pathogens and none have reported metabolic fluctuations in response to *Fol*-infection. In this research, an untargeted metabolomics approach was used to map the pattern of metabolic changes in lentil’s roots and shoots in response to *Fol*-inoculation. This study placed the emphasis on comparatively assessing the *Fol*-attributed metabolic adjustments in two contrasting genotypes, resistant and susceptible, towards discovering key metabolites that govern lentil’s response to *Fol* for possible exploitation in relevant breeding procedures. 

From an overall view, *Fol*-inoculation substantially affected the global metabolic profiles of both genotypes, as evidenced by the differences between *Fol*-inoculated and control seedlings, thus confirming that *Fol* alters lentil’s metabolism after infection. Notably, the analyses revealed that the regulation of metabolism upon *Fol*-inoculation is subjected to genotypic dependency, leading to differences in the relative abundance of specific metabolic compounds between genotypes. Addressing the observed differential response of contrasting genotypes, although there is a lack of data regarding lentil’s metabolic adjustments to different *Fol*-inoculum densities, the inoculum density employed in this study is optimal in terms of revealing genotype differences, as evidenced by previous findings related to inoculum density thresholds in resistant and susceptible genotypes [4,7]. In this context, it has been proven that under laboratory conditions, wilt incidence increases with an inoculum density above 10^3^ spores mL^−1^ and 10^5^ spores mL^−1^ in a susceptible and resistant genotype, respectively [4].

A total of 178 metabolites were detected, the vast majority of them belonging to the classes of carbohydrates, amino acids, organic acids, polyols, nitrogenous compounds and phosphate compounds. Following data processing, statistical analysis revealed significant differences in the content of 18 metabolic compounds, the differences pertaining to genotypes and/or treatments and/or their interactions. 

In relation to the metabolic profiles of carbohydrates, the analyses revealed significant differences between genotypes in the content of cellobiose, leucrose, D-trehalose and talose, the latter further differing at the treatment and at the G × T level. Such findings most likely relate to the role of carbohydrates as elicitors of plant defenses or signaling molecules in plant–microbe interactions [42], their altered content being a key feature in metabolic-based host adaptations during infection via the regulation of energy metabolism [43,44,45]. In this context, the observed increased content of both D-trehalose and cellobiose in the roots of *Fol*-inoculated seedlings of ILL-590 is relative. Trehalose, belonging to the so-called “small sugars”, is generally viewed as an essential contributor to plant defense responses to both biotic and abiotic stress factors, such as bacteria [46], fungi [47,48], aphids [49] and salinity [40]. During abiotic stress, trehalose acts as a protectant of proteins and membrane structures, an inhibitor of photo-oxidation and a key antioxidant component owing to its ROS scavenging properties [50,51,52], while in plant interactions with pathogenic or symbiotic microorganisms and herbivorous insects trehalose plays a pivotal role as a signal metabolite [52]. Considering the aspects of trehalose metabolism, its increased content specifically in the roots of *Fol*-inoculated seedlings of the resistant variety (ILL-590) supports a previously reported pattern of organ- and variety-dependent accumulation [40]. A comparable pattern of increased accumulation in infected roots of ILL-590 was noted for cellobiose, a cellulose-derived oligomer that has been shown to act as a damage-associated molecular pattern (DAMP) to elicit plant defense responses [53,54,55]. During plant–fungal interactions, the perception of molecular stress signaling from microorganisms (PAMPs) by guard cells leads to the induction of general defense responses and activation of the surveillance system to integrate signals that indicate the presence of pathogens [56,57,58,59,60]. On the other hand, pathogenic counterparts possess a range of cell wall-degrading enzymes, capable of breaking down cellulose, which acts as a physical barrier to pathogen attack, to release cellulose-derived oligomers, including cellobiose [61]. The latter, apart from acting as a DAMP-elicitor of plant defense responses [54,55,62], has been proposed as an independent activator of signaling cascade, contributing to early warning of pathogen attack [53,63]. 

Further, significant fluctuations were noted in the content of amino acids, whose metabolism is vital for immune signaling within a plant during the establishment of an immune reaction [64]. In particular, significant alterations referred to L-proline, L-asparagine and L-ornithine, the former two presenting increased content in shoots and the latter in roots. It is well known that plant–microbe interactions are interlinked with adjustments in plant nitrogen metabolism, reflecting the counteracting effects of the plant’s defense responses and the pathogen’s nutrient-starvation that may be addressed by nutrients supplied by the host [45,64,65,66]. Several lines of evidence indicate that plant–microbe interactions are marked by the metabolic disturbance of amino acids, thus highlighting their crucial role in biotic stress signaling and the establishment of a systemic immune reaction [35,45,64,67]. In this context, *Fol*-inoculation altered the content of L-proline in both shoots and roots, yet a variety-dependent metabolite response was noted, involving increased and decreased content in *Fol*-inoculated seedlings of ILL-590 and ILL-6031, respectively. The observed differential accumulation pattern is supportive of previous suggestions that proline may serve as a marker for resistance in various plant–microbe interactions, including the Fusarium head blight–cereals pathosystems, presumably due to its osmolytic features that offer protection to cellular structures [68]. Indeed, proline, apart from being a protein-forming amino acid, has been attributed complex roles in a series of cellular processes, including osmotic and redox balance, energy status, nutrient availability, cell signaling and triggering defense responses [69,70,71]. In higher plants, proline content is regulated by its biosynthesis from glutamate and its catabolism that yields glutamate as an end product, yet plant–microbe interactions may activate both pathways [70,72,73,74]. In addition to proline, the response to *Fol*-inoculation involved a marked increase in the accumulation of L-asparagine in both genotypes, mounting up to 34 fold in roots (ILL-6031) and 23 fold in shoots (ILL-590). Apart from modulating the primary nitrogen metabolism, as a main contributor to nitrogen storage, transport and partitioning, L-asparagine plays a crucial role in stress responses [44,75]. The increased content of asparagine is correlated with enhanced resistance against pathogens [76], though several pathosystems, including those of *F. oxysporum* f. sp. *cicero*–chickpea and *S. sclerotiorum*–sunflower, showed an opposite trend of increased accumulation in susceptible genotypes [36,77]. Further, *Fol*-inoculation induced an accumulation of L-ornithine in roots of both genotypes, while in the shoots a differential pattern of increased and decreased content was noted in *Fol*-inoculated seedlings of ILL-590 and ILL-6031, respectively. Ornithine, apart from being a proline precursor, consists of an intermediate product in various metabolic pathways. In this context, its accumulation pattern may relate to fluctuations observed in the content of putrescine, which is produced either directly from ornithine through the ornithine decarboxylase [78] or indirectly from arginine through the arginine decarboxylase [79]. In our study, putrescine was significantly reduced in *Fol*-inoculated seedlings of both genotypes and was depleted in *Fol*-infected roots ILL-6031. Such findings are opposed to the reported cumulative pattern of putrescine in various pathosystems, including wheat–*Fusarium graminearum* [80,81,82], attributing to putrescine and other polyamines a role as a source for ROS and stress biomarkers for stress conditions [83]. 

Significant fluctuations were also noted in compounds belonging to the class of polyols, in particular palatinitol, acetol, 2-butyne-1,4-diol and arabitol. Polyols act as multifunctional compounds, playing key roles in translocation and storage of photosynthates but also in signal transduction and stress responses, partly due to their radical scavenging properties [82,84,85]. Apart from serving as osmotically active solutes under abiotic stress conditions, polyols form an aspect of the plant response to fungal pathogens and their increased content is associated with defense, presumably acting in favor of ROS scavenging [85,86,87,88,89]. In this context, the increased accumulation of palatinitol in roots of *Fol*-inoculated seedlings of both genotypes as well as the increased content of arabitol in *Fol*-inoculated shoots of ILL-590 is relative, the latter providing further evidence related to the possible association of polyols with resistance against fungi. 

In this study, we further used gradient boosting to build a prediction model for *Fol*-resistance in lentil by metabolic indicators. To our knowledge, this is the first study to integrate machine learning models in developing genomic tools to be used in improving *Fol*-resistance in lentil. Among compounds included in our model, palatinitol, D-trehalose and fructose emerged as the most significant contributors in roots’ metabolic response to *Fol*-inoculation, whereas in shoots the highest significance was attributed to maleamic acid, L-proline and D-mannose. Such findings, apart from providing further views of lentil–*Fol* interaction, set a foundation for their possible exploitation as candidate functional markers to screen for *Fol*-resistance. Upon validation of its predictive ability, presumably via screening a larger set of lentil genotypes, the XGBoost model may be employed as a short-cut approach to interpreting the vast and complex data derived from untargeted metabolomics. 

## 4. Materials and Methods

### 4.1. Genetic Material

The genetic material consisted of two lentil genotypes, originating from ICARDA, that were previously assessed as reference germplasm for resistance and susceptibility to fusarium wilt. As such, ILL-590 served as a *Fol*-resistant genotype [9,90,91,92], while ILL-6031 was included as a *Fol*-susceptible genotype [93].

Lentil seeds were surface-sterilized using 10% sodium hypochlorite for 5 min, under gentle agitation, and then rinsed four times in sterile dH_2_O for 5 min. Sterilized seeds were pre-germinated in petri dishes containing moistened filter papers. Germinated seeds were transferred to pots (6 × 6 × 8 cm) containing sterile perlite and grown under controlled conditions (25/18 °C day/night temperature and 16/8 h light cycle, LED lighting with a Photosynthetic Photon Flux Density (PPFD) of 12 μmol m^−2^ s^−1^) until the 4-leaf stage.

### 4.2. Fol-Inoculations and Experimental Design

*Fol*-inoculations were performed using the virulent strain *Fol*F2 (GenBank Accession N. PP882814), which was morphologically and molecularly characterized as *Fol*, based on homology analysis of *Fol* isolates found in major Greek lentil cultivation zones with nucleotide sequences available in the NCBI database [94]. The inoculum was prepared by 10-day monoconidial *Fol*F2 culture grown in petri dishes containing potato dextrose agar (PDA, pH 5.5) under controlled conditions (at 26 ± 2 °C and 12/12 h light cycle). At the end of the incubation period, 5 mL dH_2_O was added into petri dishes and the supernatants were filtered through three layers of cheesecloth. The number of conidia was counted using a hematocytometer.

The inoculation of lentil seedlings was performed at their 4-leaf stage, 14 days after germination, by immersing the roots in a spore suspension adjusted at a concentration of 10^5^ spores mL^−1^, as described by Pouralibaba et al. [15], with minor modifications. Briefly, the roots of young seedlings were immersed in a spore suspension for 10 min (ILL-590-*Fol* and ILL-6031-*Fol*), whereas mock-inoculated roots were dipped in ddH_2_O, serving as controls (ILL-590-C and ILL-6031-C). All seedlings were subsequently transferred to pots (6 × 6 × 8 cm) containing sterile perlite. Seedlings were irrigated with 15 mL of Hoagland nutrient solution (Ca(NO_3_)_2_.4H_2_O:KH_2_PO_4_:KNO_3_:MgSO_4_ (5:1:5:2 mM): 7H_2_O: FeEDTA (5 μM)) and were grown under controlled conditions (25/18 °C day/night temperature and 16/8 h light cycle, LED lighting with a Photosynthetic Photon Flux Density (PPFD) of 12 μmol m^−2^ s^−1^) for a period of 14 days. Pots were irrigated two times per week. 

The experimental layout was that of a complete random design with four replications for each genotype–treatment combination (ILL-590-C, ILL-590-*Fol*, ILL-6031-C, ILL-6031-*Fol*). Sampling was performed 14 days after inoculation. Root and shoot samples from mock-inoculated (control) and *Fol*-inoculated seedlings were simultaneously collected and, following mycelium residue removal by washing in sterile ddH_2_O, were snap-frozen in liquid nitrogen and stored at −80 °C until use in the metabolomics. The absence of mycelium residues in harvested root samples was verified under a stereomicroscope. Each sample, considered as an independent biological replicate, consisted of a bulk of 4 individual seedlings.

### 4.3. Gas Chromatography–Mass Spectrometry (GC–MS)-Based Metabolomic Analysis

Sample preparation and metabolite extraction were performed using previously described methods [95]. Briefly, 50 mg ± 0.2 of root or leaf tissue was ground in liquid nitrogen, and pulverized samples were extracted in 395 μL of ice-cold methanol and 5 μL ribitol (1 mg mL^−1^), added as an internal standard. Extracts were incubated at 70 °C for 15 min under continuous shaking. Following the addition of 200 μL chloroform, samples were incubated at 37 °C for 5 min under continuous shaking. Afterwards, 400 μL of ddH_2_O was added to the extracts, and samples were vortexed and centrifuged at 13,000 rpm for 5 min. The aqueous phase (100 μL), containing the polar metabolite fraction, was transferred into new eppendorf tubes and evaporated by applying a steady stream of nitrogen gas. 

The dry residues were resuspended in 25 μL methoxyamine-HCl (MOX) (20 mg mL^−1^ in pyridine) and derivatized for 90 min at 30 °C under continuous shaking. Following centrifugation at 13,000 rpm for 30 s, 75 μL of N-methyl-N-(trimethylsilyl)-trifluoroacetamide (MSTFA, Sigma-Aldrich, Steinheim, Germany) was added, and the samples were incubated at 37 °C for 30 min. After centrifuging at 13,000 rpm for 2 min, metabolomics analysis was performed on a gas chromatographer (Agilent 7890A, Santa Clara, CA, USA) coupled to a mass spectrometer (Agilent 5973C, Santa Clara, CA, USA). The analyzer was equipped with an HP-5MS column (30 m, 0.25 mm ID, film thickness 0.25 μm, Agilent) and helium was used as the carrier gas (1 mL min^−1^). A FAME mix (Sigma-Aldrich, Steinheim, Germany) was separately injected, for the determination of retention indexes (RIs), using the pulsed split mode of injection with an injection volume of 1 μL. The GC inlet temperature was 230 °C, and total run time was 61 min. The temperature program included an initial cycle of 80 °C for 3 min, followed by an increase rate of 5 °C /min up to 320 °C and holding for 10 min. Mass spectra were generated by scanning in the *m*/*z* range 50–550, and acquisition frequency was 50 Hz. For each genotype–treatment combination, four independent biological replicates, each referring to a bulk of 4 individual seedlings, were analyzed. Blank samples were also included. 

The GC-MS data were analyzed with the AMDIS (Automated Mass Spectral Deconvolution & Identification System) software (version 2.72) [96]. The identification of metabolic compounds was performed using the Feihn libraries [97], according to their mass spectrum and retention time. The compound levels were calculated as the relative response ratio of the peak areas of the target metabolite related to the peak area of the reference metabolite (ribitol, *m*/*z* 217, RT 23.164) and normalized with respect to the sample fresh weight.

### 4.4. Statistical Analysis

Statistical analysis was performed using R, version 4.0.0 (The R Foundation for Statistical Computing, Vienna, Austria). Initially, data were filtered and 40 metabolites were excluded from data analysis based on the “80% rule”, referring to a non-missing value for at least 80% of samples. Following data pre-processing, univariate analysis was performed via two-way analysis of variance (ANOVA) using the model that contains the genotype and treatment as main factors, along with their interaction. Significant terms were examined for the root and shoot samples separately (Tables S1 and S2). FDR type I error adjustment was performed using the Benjamini and Hochberg [98] method. Metabolites with adjusted *p*-value < 0.05 were considered for the next stage of the analysis. 

Data were further subjected to multivariate analyses, specifically cluster analysis and partial least squares–discriminant analysis (PLS-DA), using the mixOmics package [99], to discover defining characteristics between root and shoot metabolic profiles of control and *Fol*-inoculated seedlings but also between ILL-590 and ILL-6031. Heat maps were constructed to visualize correlations between different classes of variables, using the median of 4 replicates for each genotype–treatment combination (*n* = 4). Metabolite features were projected on the heat map and used for sample clustering. The scaled expression value of each feature was plotted in a red–blue color scale, with red and blue colors indicating accumulated and depleted content, respectively. Hierarchical clustering analysis (HCA) was performed using Euclidean distance as a distance measure.

Finally, the XGBoost package was used for the construction of a prediction model which consisted of the variables that contribute strongly to prediction based on random forest techniques [100]. A two-level tree was used, while the number of threads and eta were set to 2 and 0.5, respectively (eta = step size shrinkage used in update to prevent overfitting). After each boosting step, the weights of the new features may be directly obtained and eta shrinks the feature weights to make the boosting process more conservative. In this study, 4 classes for prediction (ILL-590-C, ILL-590-*Fol*, ILL-6031-C, ILL-6031-*Fol*) were used. 

## 5. Conclusions

Although breeding for crop resistance is undoubtedly the most sustainable approach to coping with diseases, success in developing resistant cultivars is seriously hampered by the lack of fundamental understanding of host–pathogen interactions and mechanisms governing immunity. Addressing the goal of enhancing disease resistance in plants, advances in omics technologies offer possibilities of integrating novel tools and strategies, thus accelerating resistance breeding in various pathosystems. Breeding efforts to achieve *Fol*-resistance in lentil have so far engaged conventional approaches, and there is a research gap in the interpretation of metabolic pathways and key regulators for resistance. In our study, the comparative metabolic profiling of contrasting genotypes, with reference to their resistance to *Fol*, pinpointed key metabolic fluctuations in response to *Fol*-inoculation. Apart from changes in a good number of metabolites, overall data revealed the crucial contribution of palatinitol and L-proline in the metabolic response of roots and shoots, respectively. More importantly, L-proline exhibited an opposite accumulation pattern in *Fol*-inoculated plants of contrasting genotypes, involving increased content in the resistant genotype, therefore suggesting its possible exploitation as a biomarker for *Fol*-resistance. This study is an initial attempt to unravel lentil’s metabolic response to *Fol*, thus providing novel information related to key aspects of lentil–*Fol* interaction. Future research directions include a dynamic depiction of the metabolic state during *Fol*-infection progress, presumably also capturing the characteristic features of the secondary metabolism. Such metabolomics approaches will undoubtedly advance our understanding of lentil–*Fol* interaction and provide valuable targets for biomarker discovery, thus paving the way towards upgrading breeding efforts to tackle fusarium wilt in lentil.

## Figures and Tables

**Table 1:** Lentil’s response to *Fol*-inoculation based on the chemical groups of metabolic compounds that were significantly affected. The significance of changes was determined on the basis of the *p*-value criterion (*p* < 0.05 are marked in bold) at the level of genotype (G) and/or treatment (T) and/or G × T interaction. Metabolite content for all metabolic compounds was expressed as a ratio of *Fol*-inoculated seedlings to controls (*Fol*/C). The ratio was calculated based on the median of 4 replications of *Fol*-inoculated seedlings to the median of 4 replications of control seedlings. Significant differences (*p*-value < 0.05) are indicated in bold. For each metabolic compound, GC-MS characteristics (fragment mass, RT, retention time) and response ratios are presented.

			Ratio *Fol*/C				*p*					
			Roots		Shoots		Root			Shoot		
Metabolite	RT	Fragment Ion *m*/*z*	ILL-590	ILL-6031	ILL-590	ILL-6031	G	T	G × T	G	T	G × T
**Carbohydrates**												
Cellobiose	41.71	204	2.650	0.656	0.556	3.603	**0.032**	0.947	0.399	0.683	0.566	0.615
Leucrose	41.06	361	#	0.000	0.000	0.000	**0.028**	0.111	0.112	0.720	0.507	0.876
D-trehalose	41.05	361	2.999	1.063	0.814	0.692	**0.021**	0.354	0.402	0.455	0.865	0.876
Talose	27.13	319	#	0.000	0.000	0.000	**0.005**	**0.005**	**0.010**	0.720	0.507	0.876
**Amino acids**												
L-proline 2	12.44	142	3.946	0.284	2.266	0.468	0.170	0.328	0.380	**0.044**	0.561	0.615
L-asparagine	20.86	188	11.250	33.646	23.109	5.416	0.547	0.111	0.399	0.088	**0.010**	0.221
L-ornithine	20.37	142	##	##	##	0.642	0.437	**0.029**	0.399	0.720	0.845	0.615
**Polyols**												
Palatinitol	43.15	361	2.478	2.036	0.773	1.345	**0.000**	**0.017**	0.064	0.777	0.868	0.876
Acetol *	23.96/43.79	217	0.000 ^1^	0.000	#	0.355	0.564	0.275	0.555	**0.020**	0.561	0.615
2-butyne-1,4-diol	27.75	147	0.000	0.000	#	0.344	0.654	0.275	0.672	**0.011**	0.363	0.615
Arabitol	23.06	217	1.173	0.269	4.132	1.118	**0.047**	0.947	0.650	0.683	0.561	0.876
**Organic acids**												
Maleamic acid	11.94	244	0.715	0.386	0.499	0.840	**0.032**	**0.019**	0.080	0.192	0.445	0.615
Fumaric acid	13.71	245	#	0.000	#	0.002	-	-	-	**0.002**	**0.002**	**0.002**
Inimodiacetic acid	18.08	232	0.049	0.007	0.817	0.524	0.564	0.302	0.536	**0.036**	0.619	0.705
**Carboxylic acids**												
3-hydroxypropanoic acid	15.58	219	0.000	0.000	#	0.000	0.547	0.275	0.509	**0.004**	**0.003**	**0.004**
**Nitrogenous compounds**												
2-amino-1-phenylethanol	11.67	174	12.346	7.249	19.689	2.500	0.437	**0.019**	0.399	0.173	0.343	0.495
Putrescine	22.95	174	0.392	0.000	0.500	0.517	**0.032**	**0.001**	**0.017**	0.911	0.561	0.876
**Phosphate compounds**												
Glycerol 1-phosphate	23.87	357	1.983	0.360	##	1.332	0.642	0.769	0.100	0.182	**0.002**	0.071

* Sum of acetol 3 and 4. # Zero accumulation in *Fol*-inoculated and control seedlings. ## Zero accumulation in control seedlings. ^1^ Zero accumulation in *Fol*-inoculated seedlings.

## Data Availability

Dataset available on request from the authors.

## Supplementary Materials

The following supporting information can be downloaded at: https://www.mdpi.com/article/10.3390/plants13142005/s1, Table S1: Two-way analysis of variance (ANOVA), using the model that contains the genotype and treatment as main factors along with their interaction, for metabolites detected in roots of control seedlings and *Fol*-inoculated seedlings of ILL-590 and ILL-6031; Table S2: Two-way analysis of variance (ANOVA), using the model that contains the genotype and treatment as main factors along with their interaction, for metabolites detected in shoots of control seedlings and *Fol*-inoculated seedlings of ILL-590 and ILL-6031; Table S3: Most important contributors, according to the gradient boosting methodology.
