# Supplementary material for: Metabolic Aspects of Lentil–Fusarium Interactions
Source: Plants (Basel). 2024 Jul 22;13(14):2005. doi: 10.3390/plants13142005 (PMC11281263; doi:10.3390/plants13142005)
Supplement: Supplementary file 1 [file plants-13-02005-s001.zip › plants-3081141-supplementary.pdf]

Table S1: Two-way analysis of variance (ANOVA), using the model that contains the genotype and treatment as main factors along with their interaction, for metabolites detected in roots of control seedlings and *Fol*-inoculated seedlings of ILL-590 and ILL-6031.

N.	Metabolic compound	Genotype	Treatment	G x T
1	cellobiose	0,031980038	0,946664151	0,399395877
2	5-aminovaleric acid 1,2	0,436641999	0,301533654	0,399395877
3	L-proline 1	0,5775136	0,574930455	0,462874021
4	L-proline 2	0,170337968	0,328005487	0,380231202
5	lactobionic acid	0,170337968	0,946664151	0,932499843
6	D-mannose	0,170337968	0,514402189	0,434924346
7	maltotriose	0,544742954	0,301533654	0,976194804
8	allantoin	0,436641999	0,275186751	0,284873035
9	D (+) galactose	0,547600822	0,513538326	0,399395877
10	L-norleucine	0,436641999	0,301533654	0,380231202
11	L-asparagine	0,547303529	0,110794708	0,399395877
12	N-methylglutamic acid	0,809236824	0,610226302	0,452562527
13	tagatose	0,933014061	0,991066751	0,284873035
14	palatinitol	0,00042944	0,017228526	0,064245863
15	L-glutamic acid	0,645979121	0,542222262	0,377573554
16	isomaltose	0,170337968	0,500195599	0,665384931
17	pipecolic acid	0,564169709	0,301533654	0,399395877
18	D-glucose-6-phosphate 1,2	0,645979121	0,301533654	0,536313393
19	melibiose	0,380001131	0,110794708	0,380001131
20	D (+)altrose	0,467251819	0,611983799	0,399395877
21	talose	0,005175901	0,005175901	0,010351801
22	D-allose	0,467251819	0,83443956	0,536313393
23	D-sphingosine	0,505871638	0,511595955	0,284873035
24	maleamic acid	0,031980038	0,01868839	0,079508149
25	L-alanine	0,467251819	0,574930455	0,592331275
26	L-serine	0,547600822	0,83443956	0,284873035
27	aspartic acid	0,686966348	0,83443956	0,079508149
28	maltose	0,653790453	0,500195599	0,377573554
29	L-ornithine	0,436641999	0,0290796	0,399395877
30	L-valine	0,331992131	0,301533654	0,380231202
31	L-threonine	0,547600822	0,354012586	0,380231202
32	L-tryptophan	0,613858849	0,611983799	0,457002946
33	norvaline	0,436641999	0,661398081	0,498114997
34	L-glutamine	0,933014061	0,325650394	0,649479656
35	DL-isoleucine	0,547303529	0,445907265	0,536313393
36	acetol 1	0,642075367	0,275186751	0,649479656
37	acetol 3,4	0,564169709	0,275186751	0,554648413
38	dehydroascorbic acid	0,467251819	0,768621286	0,463914937
39	lactose	0,392135633	0,301533654	0,490337191
40	malonic acid	0,071023019	0,946664151	0,932499843
41	2-amino-1-phenylethanol	0,436641999	0,01868839	0,399395877
42	phosphoric acid	0,170337968	0,354012586	0,399395877
43	putrescine	0,031980038	0,001033888	0,016461604
44	spermidine	0,688834755	0,504775627	0,380231202
45	6-hydroxy caproic acid	0,564169709	0,574930455	0,284873035
46	D-lyxosylamine	0,222987725	0,882951915	0,857724718
47	leucrose	0,027582742	0,110794708	0,111612236
48	3-hydroxy-3-methylglutaric acid	0,564169709	0,514402189	0,508877021
49	tetratriacontane	0,436641999	0,328005487	0,976194804
50	mucic acid	0,436641999	0,325650394	0,079508149
51	6-deoxy-D-glucose	0,436641999	0,83443956	0,649479656
52	citramalic acid	0,783922553	0,91002313	0,650119098
53	trans-aconitic acid	0,467251819	0,84050914	0,699963118
54	methylmalonic acid	0,485879131	0,500195599	0,399395877
55	fructose	0,467251819	0,337629382	0,377573554

56	L-tyrosine	0,467251819	0,301533654	0,402181113
57	p-toluenesulfonic acid	0,467251819	0,301533654	0,399395877
58	phenyl-beta-glucopyranoside	0,436641999	0,328005487	0,780834975
59	3-hydroxypropanoic acid	0,547303529	0,275186751	0,508877021
60	carbazole	0,564469712	0,602777107	0,649479656
61	L-pyroglutamic acid	0,564469712	0,493686837	0,571447057
62	D-(+) trehalose	0,02085462	0,354012586	0,402181113
63	glycerol 1-phosphate	0,642075367	0,768621286	0,100337553
64	hydroquinone	0,976567245	0,500195599	0,780834975
65	2-butyne-1,4-diol	0,653790453	0,275186751	0,671694293
66	behenic acid	0,653790453	0,513538326	0,677663573
67	shikimic acid	0,919927157	0,301533654	0,299905046
68	iminodiacetic acid	0,564169709	0,301533654	0,536313393
69	melezitose	0,436641999	0,514402189	0,399395877
70	arabitol	0,04669938	0,946664151	0,649479656
71	methyl-beta-D-galactopyranoside	0,436641999	0,29641871	0,399395877
72	ribose	0,685982352	0,514402189	0,402181113
73	2-amino-2-methyl-1,3-propanediol	0,564169709	0,354012586	0,932499843
74	Beta- alanine	0,083194584	0,991066751	0,571447057
75	D-lyxose 2	0,436641999	0,301533654	0,399395877

Raw data were pre-processed based on the ’80 % rule’, referring to a non-missing value for at least 80 % of samples.

Table S2: Two-way analysis of variance (ANOVA), using the model that contains the genotype and treatment as main factors along with their interaction, for metabolites detected in shoots of control seedlings and *Fol*-inoculated seedlings of ILL-590 and ILL-6031.

N.	Metabolic compound	Genotype	Treatment	G x T
1	glucoheptonic acid	0,195812907	0,95959498	0,959754256
2	cellobiose	0,682560367	0,566158692	0,615216494
3	ribonic acid-gamma-lactone	0,244599004	0,566158692	0,630862543
4	5-aminovaleric acid 1,2	0,173328549	0,570584699	0,633982999
5	L-proline 1	0,088178654	0,147409532	0,071445173
6	L-proline 2	0,044147594	0,56052264	0,615216494
7	lactobionic acid	0,173328549	0,566158692	0,630862543
8	D-mannose	0,173328549	0,147409532	0,071445173
9	maltotriose	0,244599004	0,944146111	0,921257225
10	allantoin	0,948185891	0,497430011	0,615216494
11	D (+) galactose	0,840416351	0,95959498	0,786910744
12	L-norleucine	0,551071799	0,362946017	0,89361845
13	L-asparagine	0,088178654	0,010250096	0,220537847
14	N-methylglutamic acid	0,327009323	0,844654578	0,876377136
15	tagatose	0,445130591	0,960723821	0,921257225
16	palatinitol	0,776887162	0,868163723	0,876377136
17	L-glutamic acid	0,235721543	0,944502133	0,630862543
18	isomaltose	0,192238339	0,95959498	0,876377136
19	pipecolic acid	0,499144417	0,890051727	0,876377136
20	galactinol	0,402690673	0,95959498	0,959754256
21	D-glucose-6-phosphate 1,2	0,618843064	0,619220727	0,615216494
22	melibiose	0,925154062	0,868163723	0,630862543
23	D (+)altrose	0,615717617	0,619220727	0,959754256
24	talose	0,719601649	0,506699559	0,876377136
25	D-allose	0,533602363	0,56052264	0,472838573
26	D-sphingosine	0,875332901	0,868163723	0,921257225
27	maleamic acid	0,192238339	0,444813711	0,615216494
28	L-alanine	0,402690673	0,95959498	0,964897275
29	L-serine	0,925154062	0,56052264	0,630862543
30	aspartic acid	0,087027225	0,497430011	0,287541793
31	L-leucine	0,551071799	0,516244472	0,704919452
32	maltose	0,814262728	0,865410055	0,876377136
33	L-ornithine	0,719853615	0,844654578	0,615216494
34	L-valine	0,862720396	0,56052264	0,876377136
35	L-threonine	0,660001963	0,56052264	0,921257225
36	L-tryptophan	0,445130591	0,607097239	0,904049534
37	norvaline	0,968911052	0,566158692	0,630862543
38	gluconic acid lactone	0,151612569	0,95959498	0,959754256
39	L-glutamine	0,518040063	0,619220727	0,615216494
40	DL-isoleucine	0,647618053	0,497430011	0,921257225
41	acetol 1	0,088178654	0,985359505	0,985359505
42	acetol 3,4	0,019756897	0,56052264	0,615216494
43	dehydroascorbic acid	0,173328549	0,56052264	0,921257225
44	lactose	0,925154062	0,497430011	0,871339101
45	2-amino-1-phenylethanol	0,173328549	0,343395673	0,495403459
46	phosphoric acid	0,329921201	0,566158692	0,630862543
47	putrescine	0,911320485	0,560522640	0,876377136
48	spermidine	0,687985140	0,619220727	0,630862543
49	6-hydroxy caproic acid	0,565116879	0,657751633	0,921257225
50	D-lyxosylamine	0,925154062	0,621262639	0,615216494
51	leucrose	0,719601649	0,497430011	0,876377136
52	3-hydroxy-3-methylglutaric acid	0,456709202	0,619220727	0,871741856
53	tetratriacontane	0,530945143	0,844654578	0,319329341
54	mucic acid	0,402690673	0,959594980	0,959754256
55	6-deoxy-D-glucose	0,577553701	0,619220727	0,730845630

56	citramalic acid	0,682560367	0,497430011	0,397072500
57	trans-aconitic acid	0,173328549	0,560522640	0,876377136
58	fumaric acid	0,001632986	0,001461508	0,001775413
59	methylmalonic acid	0,719853615	0,497430011	0,654060728
60	carbamic acid ethyl ester	0,530945143	0,619220727	0,704919452
61	fructose	0,121320998	0,844654578	0,615216494
62	gluconic acid	0,308533105	0,621262639	0,876377136
63	L-tyrosine	0,445130591	0,657751633	0,704919452
64	adenosine	0,173328549	0,95959498	0,959754256
65	p-toluenesulfonic acid	0,482342861	0,95959498	0,786910744
66	L-histidine	0,072313288	0,732197324	0,871339101
67	phenyl-beta-glucopyranoside	0,088178654	0,886640123	0,615216494
68	3-hydroxypropanoic acid	0,003937483	0,002624989	0,003937483
69	carbazole	0,088178654	0,56052264	0,615216494
70	L-pyroglutamic acid	0,577553701	0,56052264	0,73084563
71	D-(+) trehalose	0,455231658	0,865410055	0,876377136
72	glycerol 1-phosphate	0,181632027	0,001461508	0,071445173
73	hydroquinone	0,682560367	0,566158692	0,876377136
74	2-butyne-1,4-diol	0,011046964	0,362946017	0,615216494
75	behenic acid	0,142615689	0,56052264	0,704919452
76	shikimic acid	0,502760183	0,566158692	0,615216494
77	iminodiacetic acid	0,036186683	0,619220727	0,704919452
78	D-(+)-melezitose	0,700586229	0,657751633	0,921257225
79	melezitose	0,968911052	0,607097239	0,876377136
80	arabitol	0,682560367	0,56052264	0,876377136
81	methyl-beta-D-galactopyranoside	0,482342861	0,638172737	0,704919452
82	ribose	0,74936356	0,855046138	0,615216494
83	2-amino-2-methyl-1,3-propanediol	0,327009323	0,732197324	0,876377136
84	Beta- alanine	0,192238339	0,95959498	0,921257225
85	D-lyxose 1	0,402690673	0,865410055	0,876377136
86	D-lyxose 2	0,746749878	0,732197324	0,630862543

Raw data were pre-processed based on the ’80 % rule’, referring to a non-missing value for at least 80 % of samples.

Table S3: Most important contributors, according to the gradient boosting methodology. Gain: relative contribution of each metabolite to the model calculated by taking each metabolite's contribution for each tree in the model. Cover: relative number of observations related to the metabolite. Frequency: percentage representing the relative number of times a particular metabolite occurs in the trees of the prediction model.

N.	Metabolic compound	Gain	Cover	Frequency
		**Roots**		
1	Patatinitol	0.500	0.500	0.500
2	D- trehalose	0.250	0.250	0.250
3	Fructose	0.250	0.250	0.250
		**Shoots**		
1	Maleamic acid	0.273	0.229	0.212
2	L-proline 1	0.262	0.201	0.182
3	D-mannose	0.258	0.199	0.182
4	Ribonic acid-g-lactone	0.071	0.046	0.030
5	Lactobionic acid	0.049	0.062	0.061
6	Aspartic acid	0.037	0.061	0.061
7	L-serine	0.018	0.032	0.030
8	L-proline 2	0.010	0.037	0.030
9	Cellobiose	0.007	0.019	0.030
10	L-threonine	0.005	0.033	0.061
11	D-glucose-6-phosphate	0.004	0.019	0.030
12	Hydroquinone	0.002	0.016	0.030
13	Phenyl-b-glycopyranoside	0.002	0.015	0.030
14	L-valine	0.002	0.021	0.030
